# State-based targeted vaccination

**DOI:** 10.1007/s41109-021-00352-z

**Published:** 2021-01-22

**Authors:** Tomer Lev, Erez Shmueli

**Affiliations:** Department of Industrial Engineering, Tel-Aviv University, Ramat Aviv, 69978 Tel-Aviv, Israel

**Keywords:** Network epidemiology, Contagion models, Targeted vaccination, State-based vaccination, Betweenness centrality

## Abstract

Vaccination has become one of the most prominent measures for preventing the spread of infectious diseases in modern times. However, mass vaccination of the population may not always be possible due to high costs, severe side effects, or shortage. Therefore, identifying individuals with a high potential of spreading the disease and targeted vaccination of these individuals is of high importance. While various strategies for identifying such individuals have been proposed in the network epidemiology literature, the vast majority of them rely solely on the network topology. In contrast, in this paper, we propose a novel targeted vaccination strategy that considers both the static network topology and the dynamic states of the network nodes over time. This allows our strategy to find the individuals with the highest potential to spread the disease at any given point in time. Extensive evaluation that we conducted over various real-world network topologies, network sizes, vaccination budgets, and parameters of the contagion model, demonstrates that the proposed strategy considerably outperforms existing state-of-the-art targeted vaccination strategies in reducing the spread of the disease. In particular, the proposed vaccination strategy further reduces the number of infected nodes by 23–99%, compared to a vaccination strategy based on Betweenness Centrality.

## Introduction

Judging by the past, infectious diseases pose one of the greatest risks for a global catastrophe. Many of these diseases (e.g., Measles, Pertussis, Influenza) have been burdening us for centuries, while new diseases (e.g., Ebola, Zika, and the recent COVID-19) continue to emerge (Morens et al. 2004).

Consequently, mathematical contagion models of diseases were developed by epidemiology researchers as a tool to study the mechanisms by which diseases spread, to predict the future course of an outbreak, and to evaluate strategies to control an epidemic (Anderson and May 1992). Traditional models assumed a fully interconnected population, in which the interactions and infections can occur between any pair of available individuals. In contrast, more recent models assume the existence of an underlying network structure that describes the potential interactions (network edges) between individuals (network nodes).

Vaccination has become one of the most prominent measures for preventing the spread of infectious diseases in modern times since it does not only protect the vaccinated individuals themselves but also prevents them from infecting their contacts (Cornforth et al. 2011). However, mass vaccination of the population may not always be possible, due to high costs, severe side effects, or shortage (Gallos et al. 2007; Vidondo et al. 2012). Therefore, identifying individuals with a high potential of spreading the disease and targeted vaccination of these individuals is of high importance (Shams 2014; Wang et al. 2016).

Various strategies for identifying such individuals have been proposed in the network epidemiology literature [for more information on vaccination and epidemics in networks the reader is referred to Wang et al. (2017b)]. The most common approach is based on vaccinating nodes with high network centrality scores. Vaccinating the highest degree node is perhaps the most studied strategy (Dezső and Barabási 2002; Schneider et al. 2012; Chen et al. 2008; Ma et al. 2013; Mao and Bian 2010; Zanette and Kuperman 2002; Vidondo et al. 2012; Holme et al. 2002), whereas, vaccinating nodes with the highest Betweenness Centrality score (Salathé and Jones 2010; Schneider et al. 2012; Hébert-Dufresne et al. 2013; Chen et al. 2008; Holme et al. 2002) is acknowledged as the most effective strategy (Wang et al. 2016). Several recent studies suggested targeted vaccination strategies that apply network centrality measures which are more “community-aware” (Cherifi et al. 2019; Ghalmane et al. 2019; Tulu et al. 2018). While this approach typically leads to high performance, it requires global information about the entire network structure, which is not always available.

In order to overcome this issue, several studies suggested a vaccination approach that requires only local information about the node’s neighborhood by relying on the friendship paradox (Feld 1991). This paradox suggests that in most cases, the friends of an individual have more friends than the individual itself. Therefore, as suggested by Cohen et al. (2003) and improved by Gallos et al. (2007), picking a random neighbor of a random node is more likely to result in a central node than just picking a random node. Such vaccination strategies are commonly called in the literature acquaintance strategies. However, since this approach uses less information than the centrality-based approach, it is typically less effective.

All of the targeted vaccination strategies described above rely solely on the network topology when choosing the nodes to vaccinate. In contrast, in this paper, we propose a novel targeted vaccination strategy, Infectious Betweenness (IB) Centrality, that considers both the static network topology and the dynamic states of the network nodes over time. This allows our strategy to find the individuals with the highest potential to spread the disease at any given point in time. More specifically, inspired by Betweenness Centrality, our strategy aims at identifying nodes that serve as bridges between network components. However, in contrast to Betweenness Centrality, our strategy focuses on finding bridges between infected nodes and susceptible nodes.

Extensive evaluation that we conducted over real-world networks demonstrates that the proposed IB Centrality strategy considerably outperforms existing state-of-the-art strategies. In particular, when compared with Betweenness Centrality (second-best strategy), IB Centrality further reduces the number of infected nodes in 23–99%. These results were robust to changes in parameters’ values, including the network topology and size, the vaccination budget, the transmission probability, and the recovery rate.

This paper is organized as follows. In “The Proposed Method” section, we present the details of the proposed targeted vaccination strategy. Then, in “Experimental setting” section, we describe the experimental setting used throughout our evaluation. In “Results” section, we report the results of our extensive evaluation. Finally, “Summary and conclusion” section summarizes this paper and suggests directions for future research.

## The Proposed Method

In this section, we present the Infectious Betweenness (IB) Centrality vaccination strategy. To that end, in “Betweenness Centrality” section, we describe the well-studied Betweenness Centrality measure, which our method is based on, and in “The SIR Model” section, we describe the susceptible-infected-recovered (SIR) model which is later used in our evaluation. Finally, in “Infectious Betweenness Centrality” section, we provide the details of IB Centrality.

**Fig. 1:**
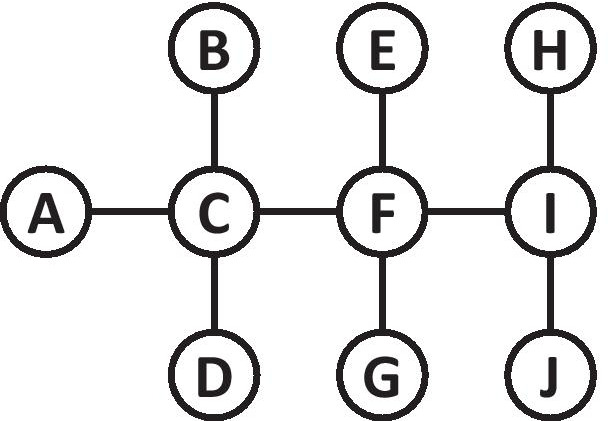
A toy network with 10 nodes and nine undirected edges

To demonstrate the ideas described in this section, we will use the toy network depicted in Fig. 1. This network includes 10 nodes connected with 9 undirected edges.

### Preliminaries

#### Betweenness centrality

The Betweenness Centrality score of a given node *v* is the fraction of shortest paths between a given pair of nodes that go through *v*, summed over all possible pairs of nodes (Freeman 1977). More formally, the Betweenness Centrality score of a node *v*, denoted by *BC*(*v*), is:

1 $$\begin{aligned} BC(v) = \sum \limits _{a, b \in V, a \ne b \ne v}\frac{\sigma (a,b|v)}{\sigma (a,b)} \end{aligned}$$

where *V* is the set of all network nodes, $\sigma (a,b)$ is the number of shortest paths that start at node *a* and end at node *b*, and $\sigma (a,b|v)$ is the number of shortest paths that start at node *a*, end at node *b* and pass through node *v*.

For example, let’s calculate *BC*(*I*) for the network shown in Fig. 1. Overall, we have 36 pairs of nodes that do not include *I* (i.e., $(10-1)(10-2) / 2$), and between each such pair of nodes in our network, we have exactly one shortest path (note that this is not necessarily the case for other networks). 15 of these pairs (i.e., the 14 pairs that include one node from $\{A,B,C,D,E,F,G\}$ and one node from $\{H,J\}$, and the additional pair (*H*, *J*)) include node *I* in the (single) shortest path, and therefore each of these pairs contribute 1 to the sum in Eq. 1. All remaining 21 pairs do not include node *I* in the (single) shortest path, and therefore each of these pairs contributes 0 to the sum in Eq. 1. Put together, $BC(I)=15$.

The Betweenness Centrality-based vaccination strategy (i.e., vaccinating the nodes with the highest Betweenness Centrality scores) is often considered the most effective targeted vaccination strategy (Wang et al. 2016). This happens since nodes with higher Betweenness Centrality scores often serve as bridges between components in the network, and their vaccination is likely to break the network into smaller unconnected components, thereby preventing the spread of the disease from one component to another.

#### The SIR model

One of the most well-studied contagion models in the epidemiological literature is the susceptible–infected–recovered (SIR) model (Kermack and McKendrick 1927). SIR was originally suggested as a compartmental model, which splits the population into three compartments: *S*—susceptible, *I*—infected, and *R*—recovered. Transitions between compartments can happen in two ways: (1) susceptible individuals have a transmission probability $\beta$ to become infected, reflecting an “interaction” with infected individuals, and (2) infected individuals recover from the disease with rate $\gamma$.

The SIR model was also studied as a network-based model [see for example (Holme 2004; Ventresca and Aleman 2013; Shaw and Schwartz 2010; Ma et al. 2013; Mao and Bian 2010; Cohen et al. 2003; Zanette and Kuperman 2002; Zuzek et al. 2015]. The contagion process, in this case, is very similar to the one in the compartmental case, but in this case, infections are restricted by the network topology. More specifically, at each timestamp, each infected node (i.e., a node with state *I*) has a single independent opportunity to infect each of its susceptible neighbors (i.e., neighbor nodes with state *S*), and this attempt succeeds with probability $\beta$. Similarly, at each timestamp, each infected node (i.e., node with state *I*) can recover (i.e., change its state to *R*) with rate $\gamma$ (or equivalently, recover after $\frac{1}{\gamma }$ time steps).

**Fig. 2:**
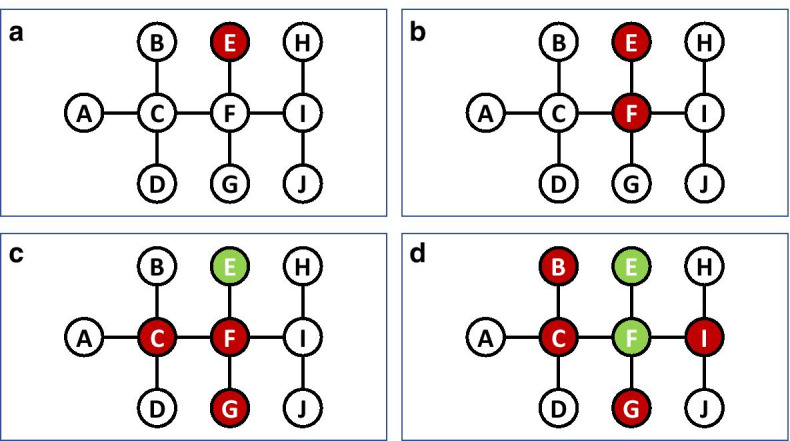
Four consecutive snapshots of the SIR contagion process over the network from Fig. 1. Susceptible nodes (*S*) are marked in white, infected nodes (*I*) are marked in red, and recovered nodes (*R*) are marked in green

Figure 2 demonstrates the contagion process according to the network-based SIR model. For that purpose, we use the same network topology from Fig. 1, and assume that $\beta =0.2$ and $\gamma =0.5$. The figure presents four consecutive snapshots of the contagion process, where susceptible nodes (*S*) are marked in white, infected nodes (*I*) are marked in red, and recovered nodes (*R*) are marked in green. At time $t=0$ (Fig. 2a), *E* is the only infected node. *E* has a single shot to infect its sole susceptible neighbor *F*, where the probability of this attempt to succeed is 0.2. Assuming that this attempt succeeded, at time $t=1$ (Fig. 2b) we have two infected nodes *E* and *F*. Now, *E* and *F* have the opportunity to infect one or more of their susceptible neighbors *C*, *G*, and *I* (each with probability 0.2). Assuming that nodes *C* and *G* got infected, and that node *E* recovered ($1/0.5=2$ timestamps have passed since its infection), at time $t=2$ (Fig. 2c) we have three infected nodes *C*, *F* and *G*. Now, *C*, *F*, and *G* have the opportunity to infect one or more of their susceptible neighbors *A*, *B*, *D*, and *I* (each with probability 0.2). Finally, assuming that nodes *B* and *I* got infected, and that node *F* recovered ($1/0.5=2$ timestamps have passed since its infection), at time $t=3$ (Fig. 2d) we have four infected nodes *B*, *C*, *G* and *I*.

### Infectious Betweenness Centrality

As mention above, most of the targeted vaccination strategies proposed in the network epidemiology literature (including Betweenness Centrality) rely solely on the network topology. In contrast, we propose a novel targeted vaccination strategy, Infectious Betweenness (IB) Centrality, that considers both the static network topology and the dynamic states of the network nodes over time. This allows our strategy to find the individuals with the highest potential to spread the disease at any given point in time.

More specifically, inspired by Betweenness Centrality, our strategy aims at identifying nodes that serve as bridges between network components. However, in contrast to Betweenness Centrality, our strategy focuses on finding bridges between infected nodes and susceptible nodes. In particular, instead of considering all shortest paths in the network, our strategy considers only the shortest paths that start with an infected node and end with a susceptible node. Formally, the IB Centrality score of a node *v* in time *t*, denoted by $IBC_t(v)$, is:

2 $$\begin{aligned} IBC_t(v) = \sum \limits _{a\in S_t, b \in I_t, a \ne b \ne v}\frac{\sigma (a,b|v)}{\sigma (a,b)} \end{aligned}$$

where $S_t$ is the set of susceptible nodes at time *t*, $I_t$ is the set of infected nodes at time *t*, $\sigma (a,b)$ is the number of shortest paths that start at node *a* and end at node *b*, and $\sigma (a,b|v)$ is the number of shortest paths that start at node *a*, end at node *b* and pass through node *v*.

**Fig. 3:**
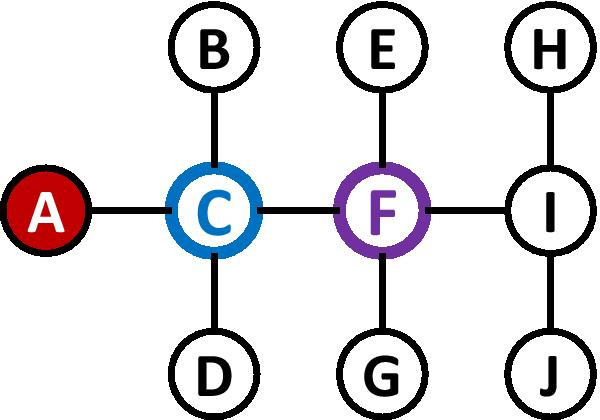
A comparison between Infectious Betweenness Centrality and Betweenness Centrality for the network from Fig. 1 and a single infected node (*A*). The node with the blue outline (*C*) has the highest Infectious Betweenness Centrality score, whereas the node with the purple outline (*F*) has the highest Betweenness Centrality score

Figure 3 demonstrates the idea behind IB Centrality. For that purpose, we use again the same network from Fig. 1. We assume an SIR contagion model, and that the only infected node at time $t=0$ is *A*. The node with the highest IB Centrality score at time $t=0$ is node *C* (marked with a blue outline), having $IBC_t(C)=8$. All 8 pairs that include node *A* (i.e., an infected node) and one node from $\{B,D,E,F,G,H,I,J\}$ (i.e., susceptible node which is not *C*) include node *C* in the (single) shortest path, and therefore each of these pairs contribute 1 to the sum in Eq. 2. Clearly, vaccinating node *C* will stop the spread of the disease completely, regardless of the values associated with $\beta$ and $\gamma$. In contrast, the node with the highest Betweenness Centrality score is *F* (marked with a purple outline), and vaccinating *F* cannot prevent the infection of nodes *B*, *C*, and *D*.

*Note* The proposed IB Centrality measure can be seen as a special case of the Q-betweenness measure (Flom et al. 2004), where the two considered groups are $S_t$ and $I_t$. It can also be seen as a special case of Betweenness Centrality defined with respect to an overlay network (Puzis et al. 2008), where the adjacency index $h_{a,b}$ is defined as:

$$\begin{aligned} h_{a,b} = {\left\{ \begin{array}{ll} 1 , &{} a \in I_t \wedge b \in S_t \\ 0, &{} \text {otherwise} \end{array}\right. } \end{aligned}$$

## Experimental setting

In this section, we describe the experimental setting used throughout our evaluation, including the network topologies used (“Network topologies” section), the contagion model considered (“Contagion model” section), the compared vaccination strategies (“Vaccination strategies” section), the simulation procedure (“Simulation procedure” section), and the parameters’ space (“Parameters' space” section).

### Network topologies

Our simulations were executed on various publicly available real-world network topologies, as detailed in Table 1. Some of the networks were adapted to fit our experimental framework, either by converting them into undirected networks or by sampling a subset of nodes.

**Table 1:** Network topologies used in our simulations

Network name	Number of nodes	Number of edges	Short description
Prison (MacRae 1960)	67	142	Friendship relationships of prison inmates
Game of Thrones (Beveridge and Chemers 2018)	407	2637	Network of interactions between characters in the HBO series “Game of Thrones”
Email (Leskovec and Krevl 2014)	1005	16,706	Email communication in a large European research institution
Facebook (Leskovec and Krevl 2014)	4039	88,234	Friendship relationships surveyed by a Facebook app
Citation (Leskovec and Krevl 2014)	3,774,768	16,518,948	US Patent citation network 1975–1999

### Contagion model

Similar to the vast majority of papers in the the network epidemiology field dealing with vaccination strategies (e.g., Ventresca and Aleman 2013; Shaw and Schwartz 2010; Ma et al. 2013; Mao and Bian 2010; Cohen et al. 2003; Zanette and Kuperman 2002; Zuzek et al. 2015), we consider the SIR contagion model. For more details, see “The SIR model” section.

### Vaccination strategies

We compare the proposed IB Centrality vaccination strategy to six other benchmark vaccination strategies:

- *Random Node* pick a random node in the network (used for example in Holme 2004; Ma et al. 2013; Shaw and Schwartz 2010).
- *Random Neighbor* pick a random node in the network and then pick a random neighbor of it (used for example in Holme 2004; Cohen et al. 2003).
- *Highest-Degree Neighbor* pick a random node in the network and then pick its highest degree neighbor (used for example in Holme 2004; Gallos et al. 2007).
- *Non-Overlap Neighbors* pick a random node *v* in the network and then pick one of its neighbors who has the highest number of neighbors that are not overlapping with *v*’s neighbors (used for example in Holme 2004).
- *Degree Centrality* pick the node with the highest Degree Centrality in the network (used for example in Dezső and Barabási 2002; Schneider et al. 2012; Chen et al. 2008; Ma et al. 2013; Mao and Bian 2010; Zanette and Kuperman 2002; Vidondo et al. 2012; Holme et al. 2002).
- *Betweenness Centrality* pick the node with the highest Betweenness Centrality in the network (used for example in Salathé and Jones 2010; Schneider et al. 2012; Hébert-Dufresne et al. 2013; Chen et al. 2008; Holme et al. 2002; Wang et al. 2016).

### Simulation procedure

In order to evaluate the proposed vaccination strategy and compare it with the benchmark strategies, we simulated the following contagion and vaccination procedure. First, a randomly selected (susceptible) node was chosen, and its state was changed to *I* in order to ignite the contagion process. Then, the process continued in rounds, where at each round, we performed the following steps: (1) applying the SIR model to allow infected nodes the opportunity to infect their susceptible neighbors as well as become recovered; (2) choosing a set of nodes to be vaccinated, according to the considered vaccination strategy and the vaccination budget constraints. (3) vaccinating the chosen set of nodes and remove them from the network. This process continues as long as the budget is not over. Once the budget is over, no more nodes are vaccinated, and the SIR model is applied iteratively until convergence (i.e., no more nodes are found in state *I*). Similar to other studies in this field (Mao and Bian 2010; Cohen et al. 2003; Zanette and Kuperman 2002; Vidondo et al. 2012; Zuzek et al. 2015), as the primary measure, we used the total number of nodes in state *R* at the end of the process (which is equivalent to the total number of nodes who were at state *I* during the contagion process).

Given a vaccination budget, *B*, we followed the methodology suggested in Wang et al. (2017a) to allocate the budget over time. More specifically, the fraction of the budget allocated at timestamp *t*, denoted by $B_t$, is given by: $B_t = B/2^t$. This allows us to allocate larger fractions of the budget at earlier stages of the contagion process, and therefore considerably limit the rate of the spread, while securing a certain fraction of the vaccination budget for later stages of the contagion process, allowing to utilize the temporal properties of the contagion process. Since our evaluation considers networks of different sizes, instead of referring to the vaccination budget in absolute numbers (denoted as *B* above), we refer to it as a fraction of the network size and denote it as *F*.

It is important to emphasize that since the states of nodes are changing over time, IB Centrality requires to recalculate the scores of susceptible nodes at each timestamp of the contagion process. This is not the case for the other benchmark strategies that can determine all nodes to be vaccinated at time $t=0$ (even if these nodes are vaccinated over time). Consequently, in principle, the benchmark strategies can choose nodes to vaccinate that are infected or recovered, and vaccinating such nodes is clearly wasteful. To make the comparison of our strategy with the benchmark strategies fairer, at each round of the simulation, we restricted the benchmark strategies to choose nodes to vaccinate from the set of susceptible nodes only.

Due to the stochastic nature of this process, each simulation was repeated 50 times, and the reported results are the average of these 50 repetitions.

### Parameters’ space

In the experiments, we examined a variety of values for the different parameters. In each set of simulations reported below, all parameters except one were set to their default value, while a single remaining parameter was examined over a varying range of values. The parameters’ space used in our experiments is detailed in Table 2.

**Table 2:** Simulation parameters space

Parameter	Values
*G*—The network topology	Prison, Game of Thrones, Email, Facebook, **Citation**
*N*—The network size	100, 500, 1000, 5000, **10,000**, 50,000, 100,000
*F*—The vaccination budget	0.01, 0.05, **0.10**, 0.15, 0.20, 0.25, 0.30, 0.35, 0.40
$\beta$—The transmission probability	0.05, 0.10, 0.15, **0.20**, 0.25
$1/\gamma$—The recovery time	**5**, 10, 15, 20, 25

Default values are marked in bold

A few of our experiments involved an analysis of varying network sizes *N*. For that purpose, we sampled the given number of nodes and the edges between them from the Citation network. To make this sampling process more robust, for each network size, we performed 30 different samplings of sub-networks of that size, and the reported results are averages over these 30 sub-networks.

## Results

In this section, we report the result of our extensive evaluation. We begin by providing an overall comparison of the proposed IB Centrality strategy with the other benchmarked methods (“Overall comparison of IB Centrality with the other benchmark methods” section), followed by a sensitivity analysis of the parameters (“Sensitivity analysis of the parameters” section) and runtime analysis (“Runtime” section).

### Overall comparison of IB Centrality with the other benchmark methods

In this subsection, we present an overall comparison of the IB Centrality strategy to the other six benchmark methods described in “Vaccination strategies” section.

**Fig. 4:**
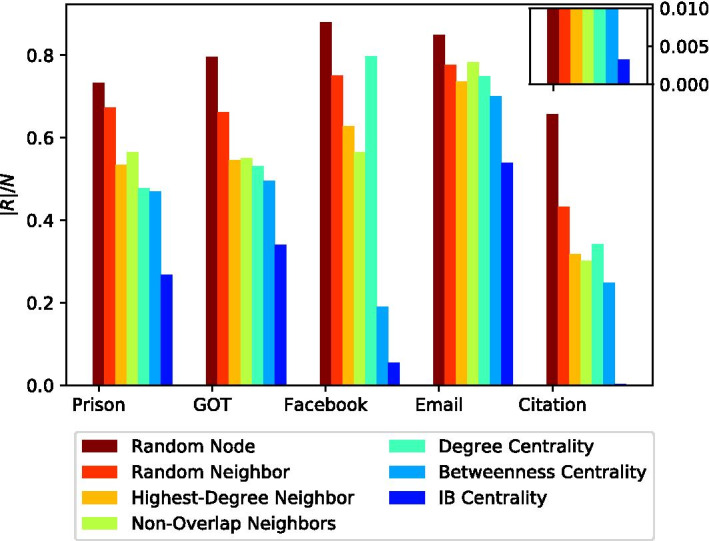
Average ratio of infected nodes after convergence |*R*|/*N* as a function of the network topology *G*. The inset on the top right corner provides a zoomed-in view of the Citation network topology. IB Centrality (blue bars) considerably outperforms all other benchmark methods

Figure 4 presents the (averaged) ratio of infected nodes after convergence |*R*|/*N*, for each of the seven considered methods, over the five network topologies described in Table 1. In this experiment, all other parameters listed in Table 2, except for the network topology, were set to their default values.

As expected, the worst-performing method is the Random Node strategy (brown bars), which does not utilize any information about the network topology nor the nodes’ states.

Next, come the Random Neighbor (red bars), Highest-Degree Neighbor (orange bars), and Non-Overlap Neighbors (green bars) strategies. As expected, these three strategies perform better than the Random Node strategy as they use some information on the network topology. It is important to note, however, that the type of information they use is restricted to the local environment of the randomly selected node.

The following are the Degree Centrality (turquoise bars) and Betweenness Centrality (light blue bars) strategies. These two strategies use global information about the network topology, which allows them a more careful selection of the nodes to vaccinate, compared to the Random Neighbor, Highest-Degree Neighbor, and Non-Overlap Neighbors strategies, which rely on local information only.

Finally, IB Centrality (blue bars) considerably outperforms all other benchmark methods. More specifically, when comparing IB Centrality to Betweenness Centrality (second-best strategy), IB Centrality obtains a ratio of infected nodes, which is 23% to 99% lower than that obtained by Betweenness Centrality. This is due to IB Centrality’s inherent ability to combine the information about the global network topology together with information about the dynamic states of nodes during the contagion process.

### Sensitivity analysis of the parameters

In this subsection, we present a comparison of the IB Centrality strategy to the other six benchmark methods for various parameters’ values that are listed in Table 2.

**Fig. 5:**
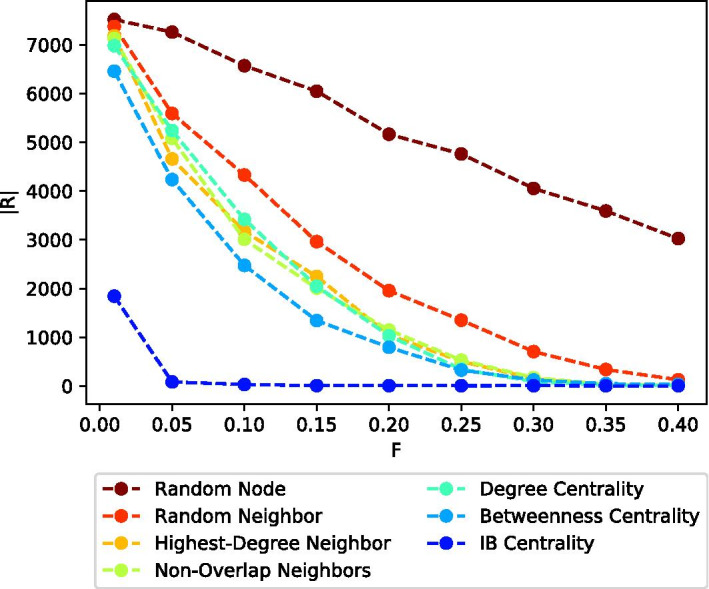
Average number of infected nodes after convergence |*R*|, as a function of the vaccination budget *F*. IB Centrality (blue plot) remains the best performing strategy for all considered values of *F*

Figure 5 presents the (averaged) total number of infected nodes (after convergence) |*R|*, for each of the seven considered methods, over the Citation network topology, for different values of the vaccination budget *F*. In this experiment, all other parameters listed in Table 2, except for *F* were set to their default values.

As expected, the number of infected nodes decreases with *F*, for all considered strategies (i.e., vaccinating more nodes reduces the number of infected nodes). However, it is interesting to see that this decrease presents a “diminishing return” effect where most of the strategies strive to zero infected nodes (for around $F\approx 40\%$). The figure also demonstrates the superiority of the IB Centrality strategy (blue plot), which remains the best performing strategy for all considered values of *F*.

**Fig. 6:**
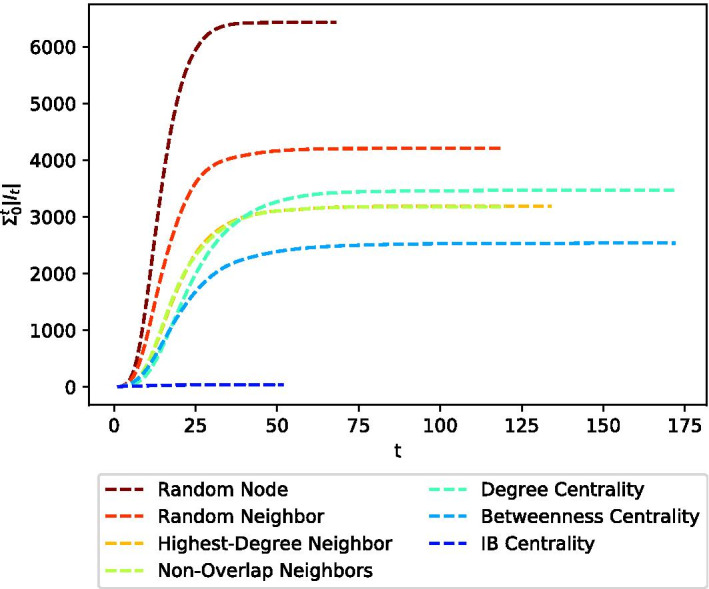
Cumulative number of infected nodes along time $\sum _0^t |I_t|$. IB Centrality maintains a seemingly “flat” curve, with a very low number of infected nodes (close to zero) during the entire contagion process

To better understand how the different vaccination strategies behave over time, we calculated the (averaged) cumulative number of infected nodes at each timestamp during the contagion process $\sum _0^t |I_t|$ (see Fig. 6), for each of the seven considered methods. In this experiment, all parameters listed in Table 2, were set to their default values. As can be seen in the figure, IB Centrality maintains a seemingly “flat” curve, with a very low number of infected nodes (close to zero) during the entire contagion process.

**Fig. 7:**
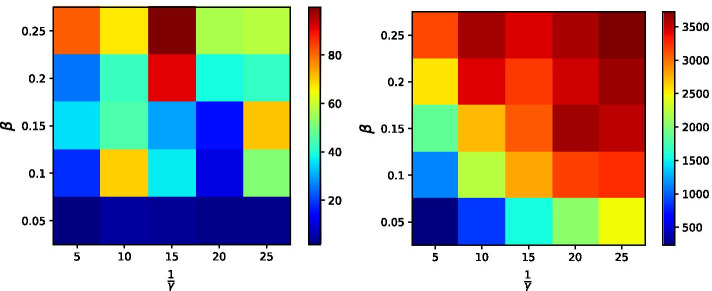
Average number of infected nodes (after convergence) |*R*|, for the IB Centrality strategy (left) and the Betweenness Centrality strategy (right), for different values of the contagion model parameters $\beta$ and $\gamma$. IB Centrality obtains |*R*| values that are considerably lower than those of Betweenness Centrality for all considered values of $\beta$ and $\gamma$

Figure 7 presents the (averaged) total number of infected nodes (after convergence) |*R*|, for the IB Centrality strategy (left) and the Betweenness Centrality strategy (right), for different values of the contagion parameters $\beta$ and $\gamma$. Each entry in the two heatmaps represents a pair of $\beta$ and $\gamma$ values, and the color of the entry represents the (averaged) total number of infected nodes (after convergence), where warmer colors represent higher |*R*| values. In this experiment, all other parameters listed in Table 2, except for $\beta$ and $\gamma$, were set to their default values.

As can be seen from the figure, IB Centrality obtains |*R*| values that are considerably lower than those of Betweenness Centrality for all considered values of $\beta$ and $\gamma$. As expected, we observe that in the case of Betweenness Centrality, |*R|* increases with higher values of the transmission probability $\beta$ and lower values of the recovery rate $\gamma$ (or higher values of $\frac{1}{\gamma }$). Interestingly, this phenomenon is not clearly evident in the case of IB Centrality. More specifically, we do see some differences between lower and higher values of $\beta$, but it is difficult to find a clear pattern with regard to $\gamma$ values. This can be a result of the relatively low values of |*R*| that make such a comparison more difficult but could also suggest that $\gamma$ has a relatively low effect on the resulting number of infected nodes in the case of IB Centrality.

**Fig. 8:**
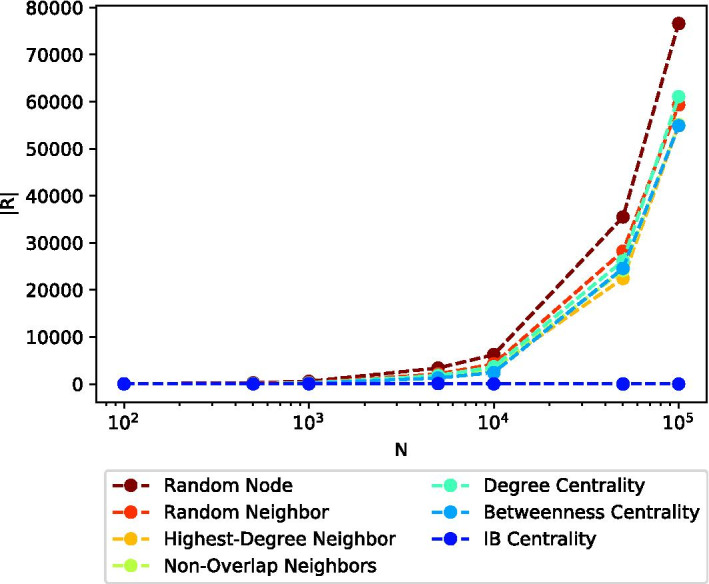
Average number of infected nodes after convergence |*R*|, as a function of the network size *N*. The X-axis (*N*) is presented in log scale. IB Centrality maintains a low number of infected nodes, even for relatively large network sizes

Figure 8 presents the (averaged) total number of infected nodes (after convergence) |*R*|, for each of the seven considered methods, and different (sampled) network sizes *N* of the Citation network topology. In this experiment, all other parameters listed in Table 2, except for *N*, were set to their default values. Again, it can be seen that IB Centrality maintains a low number of infected nodes, even for relatively large network sizes.

### Runtime

**Fig. 9:**
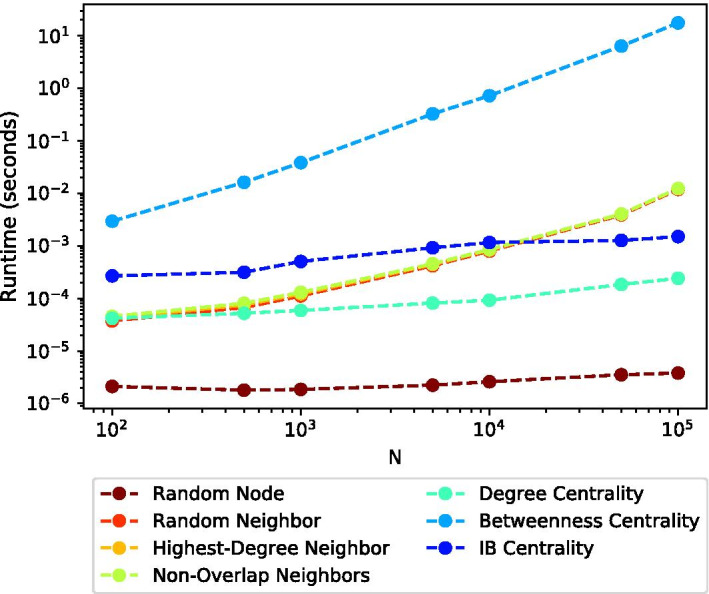
Median runtime taken to vaccinate a single node as a function of the network size *N*. Both axes are presented in log scale. IB Centrality manages to maintain a relatively fixed runtime, which is considerably better than that of Betweenness Centrality in all examined cases, and slightly better than those of the three acquaintance vaccination strategies for large network sizes

Figure 9 presents the (median) runtime taken to vaccinate a single node, for each of the seven considered methods, and different (sampled) network sizes *N* of the Citation network topology. In this experiment, all other parameters listed in Table 2, except for *N* were set to their default values.

As expected, Betweenness Centrality, which has a runtime complexity of *O*(*VE*) (Brandes 2001), takes the longest time to run for all values of *N*, and its runtime increases considerably with the network size *N* (note the log-log scale of the axes). At the other end, Random Node presents the fastest time, and this time is independent of *N*. Degree Centrality is the second-fastest strategy after Random Node, and while it involves a very simple calculation (i.e., determining the degree of a given node), it requires to perform it for all nodes in the networks, and therefore its runtime depends on *N*. Somewhere in the middle, we find the three acquaintance vaccination strategies (Random Neighbor, Highest-Degree Neighbor, and Non-Overlap Neighbors). While these strategies do not require to perform a calculation for each network node (in contrast to Degree Centrality, and similarly to Random Node), their runtime still depends on *N*. This happens since the probability of randomly picking a node having no neighbors at all or having only infected neighbors increases at later stages of the contagion process, making it more time consuming for the strategy to pick nodes to vaccinate. IB Centrality’s runtimes are slightly higher than those of the three acquaintance vaccination strategies for small network sizes. However, since it manages to maintain a relatively fixed runtime, it becomes faster for large network sizes.

## Summary and conclusion

In this paper, we proposed a novel targeted vaccination strategy, IB Centrality, which relies on both the static network topology as well as the dynamic states of the nodes during the contagion process. More specifically, at any given time *t*, the strategy recalculates scores for all susceptible nodes, where the score of a given node *v* is the fraction of shortest paths between a given pair of nodes that go through *v*, summed over all pairs of infectious nodes (at time *t*) and susceptible nodes (at time *t*). Then, depending on budget constraints, nodes with the highest scores are chosen to be vaccinated. Extensive evaluation that we conducted over a multitude of parameters’ values showed that our strategy considerably outperforms state-of-the-art vaccination strategies in all considered cases.

Additional research directions may include the following:

- *Larger Network Sizes* The network sizes considered in this paper ranged between several dozens to 100,000 nodes, which reflect small to medium real-world networks. Future research should examine settings with a considerably larger-sized network and evaluate the effectiveness as well as the efficiency of the proposed method under such settings.
- *Additional Contagion Models* In this paper, we considered the well-studied SIR model as the underlying contagion model. However, many other contagion models were proposed in the literature, including susceptible-infected-recovered-susceptible (SIRS) (Enatsu et al. 2012; Hu et al. 2011; Jin et al. 2007) and susceptible-infected-susceptible (SIS) (Hethcote 1989) to name a few. It would be interesting to investigate the properties of the proposed method under different contagion models.
- *Partial Information* The proposed strategy requires complete information on both the network topology and the states of nodes at any given time. An interesting future research direction would be to evaluate the proposed method and adapt it to settings where only partial information (on either the network topology or states of nodes) is available.
- *Closely related domains* In this paper, we focused on the targeted vaccination problem, which aims at selecting a subset of nodes to be vaccinated in order to minimize the spread of a disease.
  A closely related problem is that of influence maximization, which aims at selecting a subset of nodes to be seeded in order to maximize the diffusion of information. While the two problems are different (diffusion of information differs from a spread of diseases, and maximization is different from minimization), it would be interesting to adapt and evaluate IB Centrality in the influence maximization problem. This direction is even more compelling since several recent works that studied influence maximization suggested strategies for adaptive seeding of nodes (Goldenberg et al. 2018; Jankowski et al. 2017a, b; Sela et al. 2018).
  Another closely related domain is that of computer viruses. Similarly to biological viruses, computer viruses may spread over the computer network, and computers may be “vaccinated” by installing an antivirus software or software patches. A few studies in this domain (e.g., Chen and Carley 2004; Khouzani et al. 2011) offered dynamic patching strategies which are similar in mind to the targeted vaccination strategies discussed in this paper. An interesting research direction would be to compare strategies developed in the two domains, and adopt strategies developed in one domain to the other domain.

## Data Availability

All data used in this paper is publicly available, as described in “Network topologies” section.

## Abbreviations

IB: Infectious betweenness
SIR: Susceptible–infected–recovered
SIRS: Susceptible-infected-recovered-susceptible
SIS: Susceptible–infected–susceptible
